# Screening and verification of drugs targeting myocardial ischemia-reperfusion injury

**DOI:** 10.1097/MD.0000000000047443

**Published:** 2026-01-30

**Authors:** Qi Liu, Zhonggen Tian, Shouhu Mi, Zhen Liang, Bo Hang

**Affiliations:** a Department of Anesthesiology, Norinco General Hospital, Xi’an, China; b Department of Joint Surgery, Norinco General Hospital, Xi’an, China; c Department of Orthopedic Trauma, Norinco General Hospital, Xi’an, China.

**Keywords:** myocardial ischemia-reperfusion injury, random forest, targeted drug

## Abstract

This study focuses on the research of the molecular mechanism of myocardial ischemia-reperfusion injury (MIRI) and the exploration of treatment methods. Firstly, through the analysis of differentially expressed genes (DEGs) in 2 datasets, GSE108940 and GSE160516, 229 DEGs were identified. Further, by using the least absolute shrinkage and selection operator regression and random forest analysis, 3 key genes, *FAS*, *PPARA*, and *FGF9*, were screened out. To validate the reliability of these genes, their expression levels were observed in the merged set, experimental set, and validation set. It was found that *FAS* was highly expressed in the MIRI group, while *PPARA* and *FGF9* were lowly expressed in the MIRI group, and these results were verified in the validation set. In addition, the receiver operating characteristic curve analysis demonstrated that *FAS*, *PPARA*, and *FGF9* had good diagnostic values, with the area under the curve all >0.8. The study also explored the signaling pathways and molecular mechanisms in MIRI. The Gene Ontology analysis of the DEGs was mainly enriched in responses to interleukin-1, major histocompatibility complex protein complex, and chemokine activity. The Kyoto Encyclopedia of Genes and Genomes enrichment analysis showed that the DEGs were associated with the interaction between viral proteins and cytokines and their receptors, rheumatoid arthritis, and influenza A. The results of cell experiments showed that in the hypoxia/reoxygenation model, the cell viability decreased, and the contents of cTnI, creatine kinase, and lactate dehydrogenase were relatively high. After the administration of the candidate drugs resveratrol and GW4064, the cell viability was significantly improved, and the contents of cTnI, creatine kinase, and lactate dehydrogenase were reduced, indicating a significant effect on repairing damaged cells. Through Western blotting and immunofluorescence techniques, it was found that resveratrol and GW4064 could significantly decrease the expression of *FAS* and increase the expression of *PPARA* and *FGF9* (*P* < .01), suggesting that these drugs may have potential therapeutic effects on MIRI. Overall, this study not only provides a new perspective for understanding the molecular mechanism of MIRI but also offers valuable clues for the development of new treatment strategies.

## 1. Introduction

Acute myocardial infarction (AMI) is the most common type of coronary artery disease, primarily caused by the interruption of blood flow due to coronary artery stenosis or occlusion. The incidence and mortality rates of AMI are high worldwide, resulting in millions of deaths annually and posing a significant threat to human health.^[1,2]^ In China, the complications of AMI include heart failure, arrhythmia, coronary artery spasm, post-myocardial infarction syndrome, cardiovascular sudden death, valvular dysfunction, cerebrovascular diseases, renal insufficiency, and endocrine and metabolic disorders. These complications can severely impair patient prognosis and quality of life, emphasizing the importance of early diagnosis and comprehensive treatment. Among the numerous complications of AMI, myocardial ischemia-reperfusion injury (MIRI) is a major cause of death and heart failure.^[3–5]^ MIRI significantly impairs the cardiovascular system, ultimately leading to cardiac structural and functional deterioration and even threatening patient survival. Its effects on the cardiovascular system are extensive, including myocardial cell damage, dysfunction of cardiac contraction and relaxation, arrhythmia, expansion of infarct size, myocardial fibrosis, cardiac remodeling, endothelial dysfunction, exacerbated inflammatory responses, and heart failure, resulting in severe structural and functional damage to the heart and posing a serious threat to patient prognosis.^[6–8]^ Several key factors contribute to the progression of MIRI. The duration of ischemia is positively correlated with the severity of myocardial injury; longer ischemic periods result in more severe damage. Abnormal hemodynamic parameters and poor oxygen delivery during reperfusion further exacerbate myocardial injury. Endothelial cell damage during reperfusion disrupts vascular function. Additionally, myocardial tissue can be infiltrated by immune cells, triggering inflammatory responses. The mechanisms underlying MIRI primarily involve the following aspects: significantly increased enzyme-mediated reactions during reperfusion, directly damaging myocardial cells; a marked increase in the generation of reactive oxygen species, which attack myocardial cell membranes and nucleic acids, leading to myocardial cell destruction; infiltration of inflammatory cells and release of cytokines, further aggravating myocardial injury; calcium ion overload disrupting intracellular ion homeostasis and causing myocardial cell damage; and increased apoptosis of myocardial cells, reducing myocardial cell numbers and worsening myocardial injury.^[9,10]^ MIRI is a critical factor contributing to poor prognosis in patients with AMI. In-depth research into the mechanisms of MIRI is essential for identifying effective treatment strategies, reducing mortality, and improving the quality of life for patients.

High-throughput sequencing (HTS) is a sequencing method based on computer and automation technologies, which can sequence a large number of genes, proteins, or other biomolecules in a short period. Obtaining disease-related targets and inferring relevant targeted compounds through HTS is an important step in modern drug research and development.^[11,12]^ Therefore, in this study, we obtained targets related to MIRI from the HTS datasets of MIRI. Subsequently, machine learning algorithms were used to screen targets with potential value. We then predicted relevant targeted compounds by integrating multiple databases and verified the accuracy of the screening through cell experiments. The aim of this study is to accelerate the process of new drug development for MIRI and improve the success rate of drug research and development.

## 2. Materials and methods

### 2.1. Screening of DEGs in MIRI

The MIRI dataset was obtained from the public repository NCBI GEO (https://www.ncbi.nlm.nih.gov/geo/), with the data type based on array-based expression profiling. The selection criteria for the screening process were as follows: samples included both normal control groups and MIRI groups, and the total number of samples was >10. In this study, GSE108940 and GSE160516 were selected as the experimental datasets, and GSE58486 was used as the validation dataset. The “SVA” and “limma” packages in R software were employed to merge the aforementioned GSE108940 and GSE160516 datasets. The screening conditions were set as |log2 Fold Change|>1 and *P* < .05 to identify differentially expressed genes (DEGs).

### 2.2. Analysis of GO molecular functions and KEGG signaling pathways of DEGs

The DEGs identified in MIRI were imported into R version 4.1.2 software (https://www.r-project.org/), and gene ontology (GO) molecular function and *KEGG* pathway enrichment analyses were conducted using the ClusterProfiler and ggplot2 packages.

### 2.3. Screening of key DEGs in MIRI

Least absolute shrinkage and selection operator (LASSO) regression analysis of mRNA expression for MIRI DEGs was performed using the “glmnet” package in R software, complemented by cross-validation with the lambda selection method. Subsequently, the SVM-RFE (Support Vector Machine Recursive Feature Elimination) algorithm was applied by importing the MIRI DEGs file into the R software. Utilizing the “el071,” “kernlab,” and “caret” packages in R, cross-validation was conducted to identify the point with the minimum cross-validation error and to extract the corresponding genes. Furthermore, to mitigate overfitting, the RFE (Recursive Feature Elimination) algorithm was employed to select the optimal genes from the metadata queue. The “venn” package in R software was then used to intersect the genes identified by the SVM-RFE algorithm with those from LASSO regression, thereby filtering out the key DEGs associated with MIRI. The “pROC” package in R software was used to plot the receiver operating characteristic (ROC) curve and calculate the area under the curve to ascertain the diagnostic value of the key DEGs. Additionally, the “limma” and “ggpubr” packages in R were employed to generate Box plots representing the expression levels of the key DEGs.

### 2.4. Screening of therapeutic compounds targeting key DEGs in MIRI

A search was conducted in the comparative toxicogenomics database, the Traditional Chinese Medicine Systems Pharmacology Database and Analysis Platform (TCMSP) (https://www.tcmsp-e.com/tcmsp.php), and PubMed (https://pubmed.ncbi.nlm.nih.gov/) to identify natural compounds that could reduce or affect the key DEGs associated with MIRI. Cytoscape version 3.9.0 software was utilized to systematically visualize the results targeting natural compounds and to obtain their intersection. This process enabled the screening and selection of therapeutic compounds targeting the key DEGs in MIRI.

### 2.5. Experimental cells and reagents

Mouse myocardial H9c2 cells were purchased from Wuhan Procell Life Science & Technology Co., Ltd. (Wuhan, China). DMEM high-glucose medium and trypsin were obtained from Shanghai Beyotime Biotechnology Co., Ltd. (Shanghai, China); cell lysis buffer was purchased from Boster Biological Engineering Co., Ltd. (Wuhan, China). The rat cardiac troponin I (cTnI) enzyme-linked immunosorbent assay (ELISA) kit, rat creatine kinase (CK) activity kit, and lactate dehydrogenase (LDH) activity detection kit were all purchased from Solarbio Company (Beijing, China), with the product numbers SEKR-0048, BC1145, and BC0680, respectively. Antibodies against *FAS*, *PPARA*, *FGF9*, and β-actin, as well as HRP-labeled goat anti-rabbit IgG antibody, with batch numbers (A23810, A24835, A6374, AC026, AS080), respectively, were purchased from ABclonal Company (Wuhan, China).

### 2.6. Cell culture and hypoxia/reoxygenation models

H9c2 cells were seeded in 60-mm culture dishes and cultured in DMEM medium containing 10% fetal bovine serum (FBS) and 100 μg/mL penicillin/streptomycin. After cell synchronization, when the H9c2 cells reached a confluence of 80% to 90%, the cells were subjected to hypoxia/reoxygenation (H/R) treatment. The medium of the cells was replaced with low-glucose medium without FBS, and the cells were cultured under hypoxic conditions in a tri-gas incubator (37°C, 5% CO₂, and 1% O₂) for 12 hours. Subsequently, the medium was replaced with DMEM without FBS, and the cells were cultured under normoxic conditions in a normal incubator (37°C, 5% CO₂, and 95% air) for 12 hours. Cells that were continuously cultured in the normal incubator without medium replacement for the same duration were used as the Con group. In the H/R + resveratrol and H/R + GW4064 groups, the cells were pretreated with 50 μmol resveratrol or 1 μmol GW4064 for 2 hours before H/R treatment.

### 2.7. Tetramethyl azazole salt method

The viability of cells in vitro was evaluated using the tetramethyl azazole salt (MTT) assay. H9c2 cells were seeded in 96-well plates. After H/R or GL treatment, the cells were incubated in a medium containing MTT solution (0.5 mg/mL) with 3-(4, 5-dimethylthiazol-2-yl)–2, 5-diphenyltetrazolium bromide for 4 hours. The supernatant was discarded, and dimethyl sulfoxide (DMSO) was added. The DMSO suspension containing the cells was aspirated, and the absorbance value (optical density, OD) was quantified at 490 nm. The cell viability was quantified using the following calculation formula: cell viability = OD of the treatment group/OD of the control group.

### 2.8. Detection of myocardial injury markers

The level of cTnI was detected according to the instructions of the cTnI ELISA kit. The levels of CK and LDH were measured following the instructions of the CK activity kit and the LDH activity detection kit, respectively.

### 2.9. Western blotting

Proteins were extracted from H9c2 cells, and the protein concentration was determined using a bicinchoninic acid kit. Proteins (10–20 μg) were separated by sodium dodecyl sulfate-polyacrylamide gel electrophoresis and transferred onto a polyvinylidene fluoride membrane using an electro-transfer device. The membrane was cut and incubated with primary antibodies against *FAS* (1:1000), *PPARA* (1:1000), *FGF9* (1:1000), and β-actin (1:1000), respectively. Subsequently, the membrane that had been incubated with the primary antibodies was incubated with a goat anti-rabbit secondary antibody for 2 hours. Enhanced chemiluminescence reagents were used for visualization, and the images were saved. Finally, semiquantitative analysis of the gray values of the images was performed using ImageJ measurement software.

### 2.10. Immunofluorescence

After the cell intervention was completed, podocytes were fixed with 4% paraformaldehyde for 30 minutes, washed 3 times with PBS, and then treated with 0.5% Triton X-100 at room temperature for 20 minutes. After being washed 3 times with PBS, the cells were blocked with 3% BSA at room temperature for 30 minutes. Subsequently, the cells were incubated with the primary antibody *FAS* (1:100) at 4°C overnight. The next day, the cells were taken out and incubated with the rabbit secondary antibody at 37°C for 1 hour. Then, the cells were stained with DAPI for 5 minutes, observed under a fluorescence microscope, and the images were captured and saved.

### 2.11. Statistical method

The experimental data were analyzed using R (version 4.1.2), SPSS 25.0, and GraphPad Prism 9 statistical software. Statistical methods employed included 1-way analysis of variance (ANOVA) and *t* tests, with *P* < .05 considered to indicate a statistically significant difference.

## 3. Result

### 3.1. Screening of DEGs

**Figure 1.**
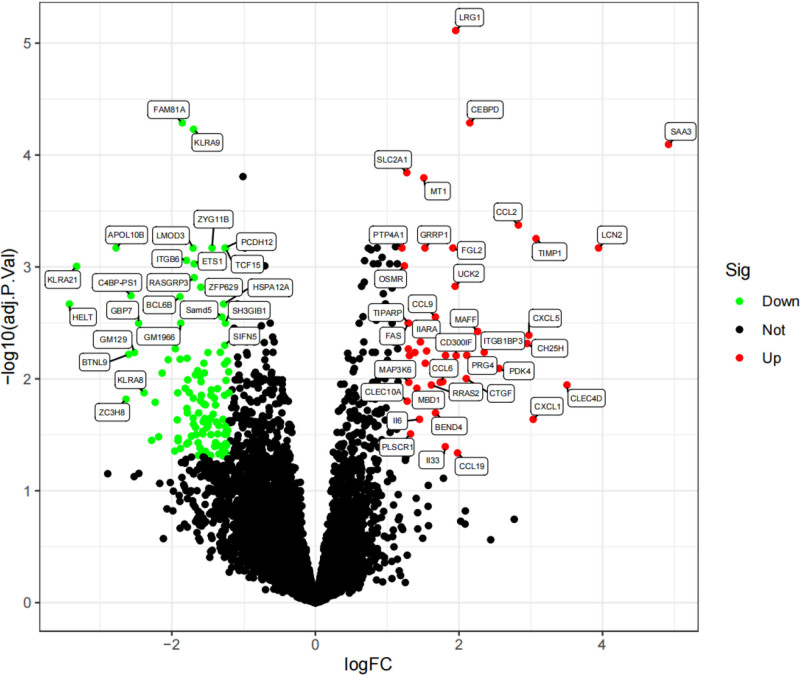
DEGs volcano map in the sham operation group and MIRI. DEGs = differentially expressed genes, MIRI = myocardial ischemia-reperfusion injury.

Differential gene analysis of the merged dataset of GSE108940 and GSE160516 revealed that there were 229 DEGs between the sham-operated group and the MIRI group. Among them, 64 genes were upregulated, and 165 genes were downregulated (Fig. 1).

### 3.2. GO and KEGG enrichment analysis

**Figure 2.**
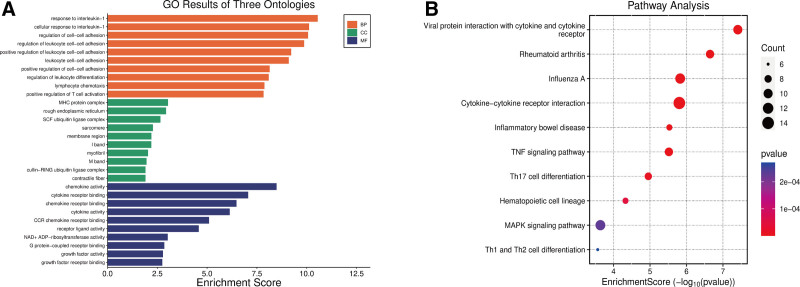
GO and *KEGG* enrichment analysis of DEGs. (A) GO enrichment analysis; (B) *KEGG* enrichment analysis. DEGs = differentially expressed genes, GO = gene ontology.

The GO analysis of the DEGs was mainly enriched in “response to interleukin-1,” “major histocompatibility complex (MHC) protein complex,” and “chemokine activity.” The *KEGG* enrichment analysis showed that the DEGs were associated with “viral protein interaction with cytokine and cytokine receptor,” “Rheumatoid arthritis,” and “Influenza A”(Fig. 2).

### 3.3. LASSO and random forest algorithm screen differential genes

**Figure 3.**
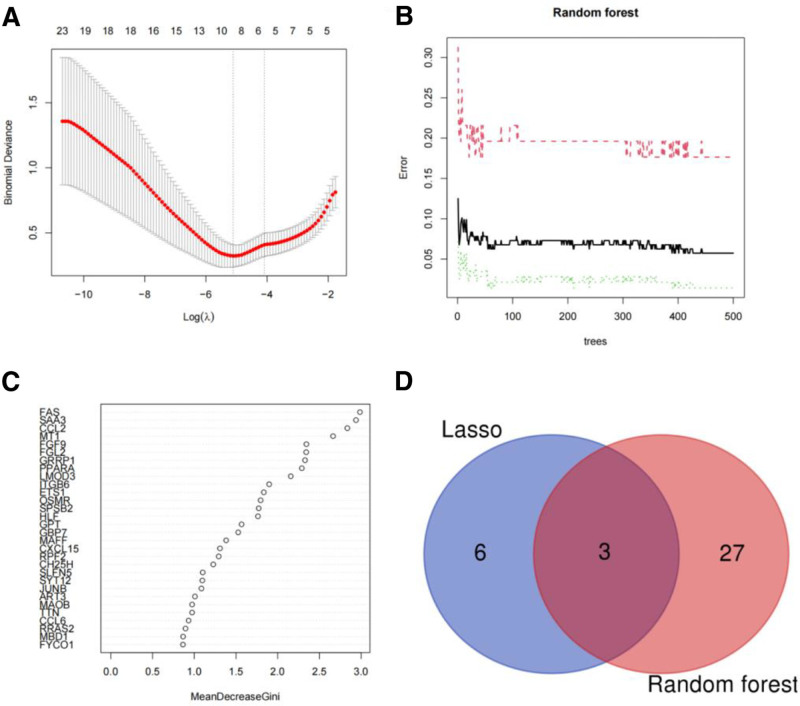
Screening of differential genes. (A) 9 DEGs were selected by LASSO regression analysis; (B, C) 30 differential genes were identified by random forest analysis; (D) intersection of LASSO and random forest screening DEGs. DEGs = differentially expressed genes, LASSO = least absolute shrinkage and selection operator.

We conducted further analysis on the DEGs. Through LASSO regression analysis, 9 genes were screened out, and 30 genes were obtained by random forest screening (Fig. 3). We then took the intersection of the genes screened by LASSO and random forest, and identified *FAS*, *PPARA*, and *FGF9*.

### 3.4. Expression of FAS, PPARA, and FGF9 genes in the dataset

**Figure 4.**
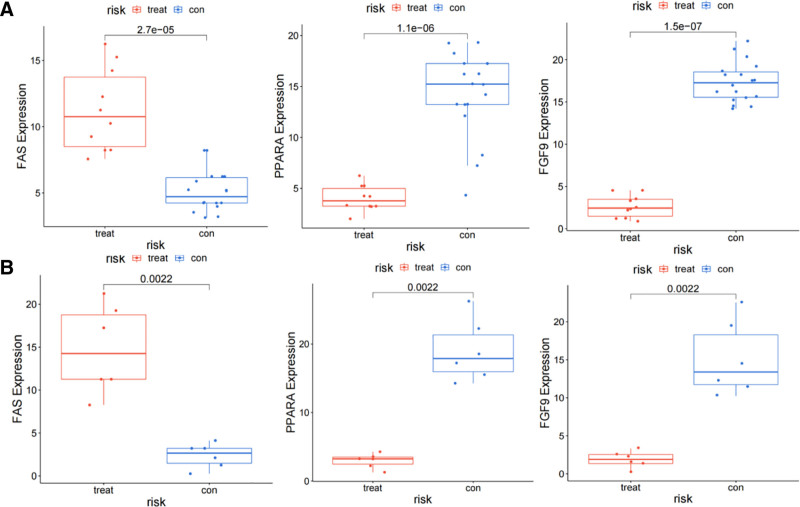
Expression of *FAS*, *PPARA*, and *FGF9*. (A) The expression of *FAS*, *PPARA,* and *FGF9* in the experimental set; (B) Verify the expression of *FAS*, *PPARA*, and *FGF9* in the set.

To further validate the reliability of the *FAS*, *PPARA*, and *FGF9* genes, we observed the expression of these genes in the merged dataset (GSE108940 and GSE160516), the experimental dataset, and the validation dataset (GSE58486), respectively. In the experimental dataset, compared with the control group, *FAS* was highly expressed in the MIRI group, while *PPARA* and *FGF9* were lowly expressed in the MIRI group (Fig. 4). Similarly, these results were also confirmed in the validation group.

### 3.5. ROC curves of FAS, PPARA, and FGF9

**Figure 5.**
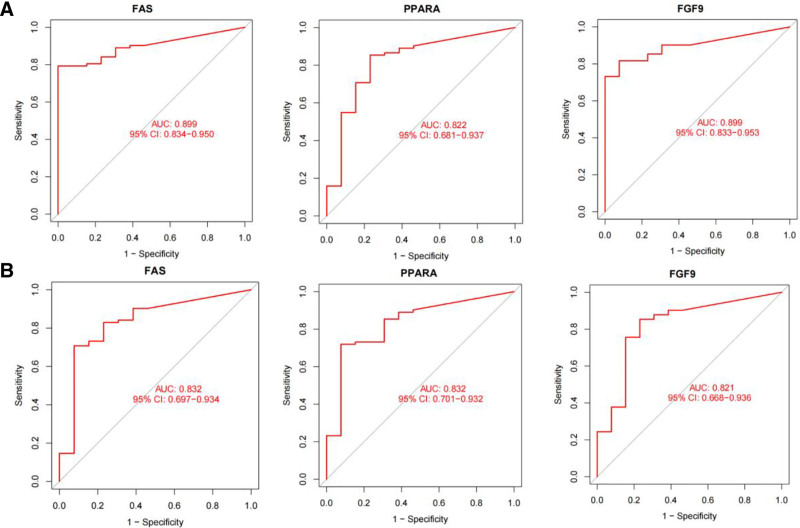
ROC curves of *FAS*, *PPARA*, and *FGF9*. (A) ROC curves of *FAS*, *PPARA* and *FGF9* in the experimental set; (B) Verify ROC curves of the set *FAS*, *PPARA*, and *FGF9*. AUC = area under the curve, ROC = receiver operating characteristic.

To further validate the diagnostic value of *FAS*, *PPARA*, and *FGF9*, we observed the area under the ROC curve of *FAS*, *PPARA*, and *FGF9* in the experimental set and the validation set, respectively. It was found that the area under the curve of *FAS*, *PPARA*, and *FGF9* was all >0.8, indicating good diagnostic value. This result was also confirmed in the validation group (Fig. 5).

### 3.6. Screening of targeted drugs

**Figure 6.**
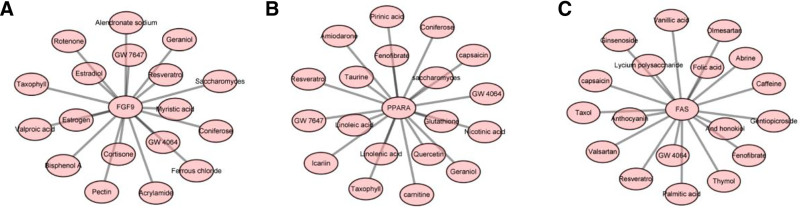
Screening of targeted drugs. (A) drugs that target *FGF9*; (B) drugs targeting *PPARA*; (C) drugs targeting *FAS*.

The comparative toxicogenomics database and TCMSP databases were used to screen drugs targeting *FAS*, *PPARA*, and *FGF9*. The screening results are as follows: 18 compounds were found to target *FAS* and *FGF9*, 19 compounds were found to target *PPARA*, and GW4064 and resveratrol were the compounds that commonly targeted *FAS*, *FGF9*, and *PPARA* (Fig. 6).

### 3.7. Effects of resveratrol and GW4064 on cardiomyocytes after hypoxic injury

**Figure 7.**
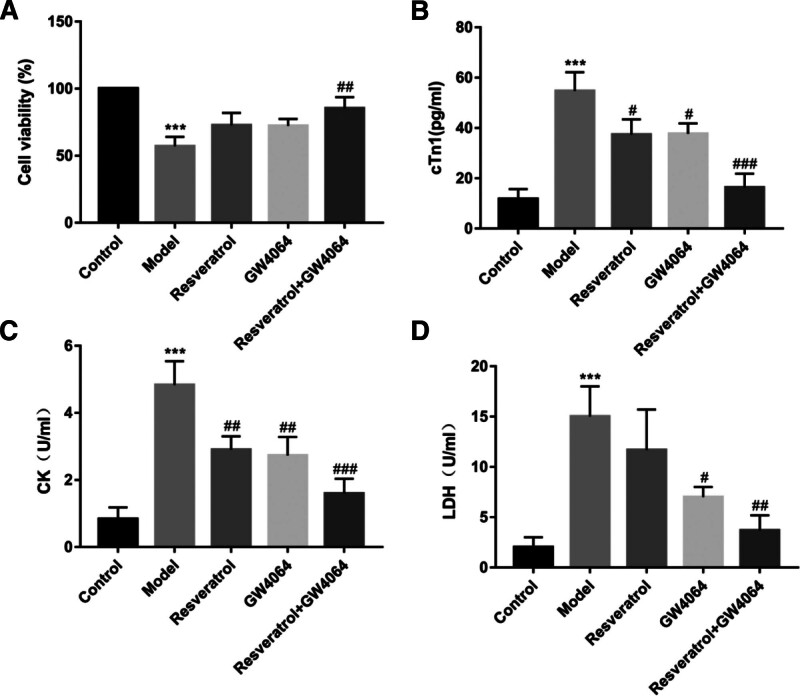
Effects of resveratrol and GW4064 on cardiomyocytes after hypoxic injury. (A) cell viability was measured by MTT; (B) The content of cTnI; (C) the content of CK; (D) LDH content; in comparison with the control group, ^***^P < .001; in comparison with the model group, ^###^*P* < .001, ^##^*P* < .01, ^#^*P* < .05. CK = creatine kinase, cTnI = cardiac troponin I, LDH = lactate dehydrogenase, MTT = tetramethyl azazole salt.

To explore the protective effects of the screened drugs, resveratrol and GW4064, on cardiomyocytes, we conducted an MTT assay and measured the levels of cTnI, CK, and LDH. The experimental results are shown in Fig. 7. In the model group, that is, the H/R model, the cells exhibited low viability. However, after the administration of resveratrol or GW4064, the viability of H9c2 cells was significantly increased (Fig. 7A). The results from the kit assays showed that the levels of cTnI, CK, and LDH in the model group were significantly higher compared with those in the control group (*P* < .001). In contrast, the levels of cTnI, CK, and LDH in the GW4064, resveratrol + GW4064 groups were significantly lower than those in the model group, and the differences were statistically significant (*P* < .05; Fig. 7, BCD).

### 3.8. The expressions of FAS, FAS, PPARA, and FGF9 were detected by Western blotting

**Figure 8.**
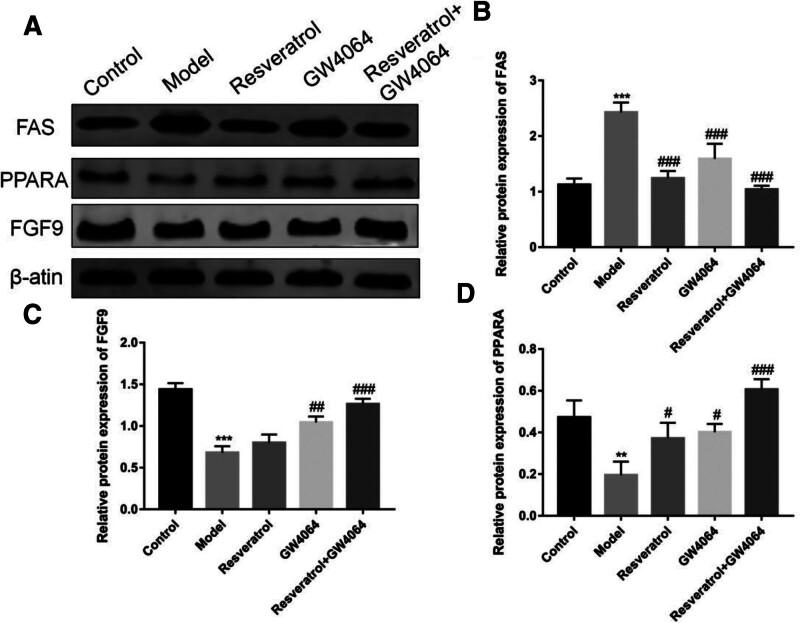
Protein expression of *FAS*, *PPARA*, and *FGF9*. (A) protein expression band map of *FAS*, *PPARA* and *FGF9*; (B) *PPARA* protein band semiquantitative statistical map; (C) semiquantitative statistical map of *FGF9* protein bands; (D) Semiquantitative statistical map of *PPARA* protein band; in comparison with the control group, ^***^P < .001, ^**^P < .01; in comparison with the model group, ^###^*P* < .001, ^##^*P* < .01, ^#^*P* < .05.

As shown in Fig. 8, compared with the control group, the expression of *FAS* in the model group was significantly increased (*P* < .001). When compared to the model group, the expression of *FAS* was significantly decreased in the resveratrol group (*P* < .001) and also notably reduced in the GW4064 group (*P* < .001). The combined use of resveratrol and GW4064 led to a further decrease in *FAS* expression. In comparison with the control group, the expression of *PPARA* in the model group was significantly reduced (*P* < .01), and the expressions of *PPARA* and *FGF9* were both markedly decreased (*P* < .01). In comparison with the model group, the expression of *PPARA* in the resveratrol group was significantly increased (*P* < .05), and it was also significantly elevated in the GW4064 group (*P* < .05). The combined use of resveratrol and GW4064 further enhanced the expression levels of *PPARA* and *FGF9* (*P *< .001).

### 3.9. The fluorescence intensity of the FAS protein was detected by immunofluorescence

**Figure 9.**
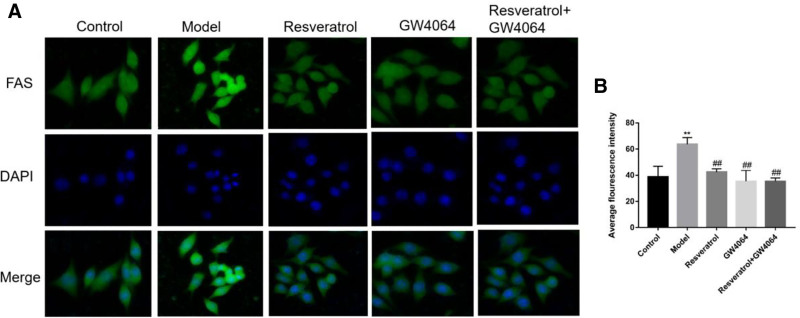
Protein fluorescence intensity of *FAS*. (A) immunofluorogram; (B) semiquantitative statistical map of fluorescence intensity; in comparison with the control group, ^**^P < .01, and in comparison with the model group, ^##^*P* < .01.

Compared with the control group, the immunofluorescence intensity of *FAS* in the model group was significantly increased (*P* < .01). Compared with the model group, the immunofluorescence intensity of *FAS* in the resveratrol group was significantly decreased (*P* < .01), and that in the GW4064 group was also significantly reduced (*P* < .01). When resveratrol was combined with GW4064, the immunofluorescence intensity of *FAS* was further reduced (*P* < .01) (Fig. 9).

## 4. Discussion

In the research on the treatment of MIRI, the development of targeted drugs has become a hot topic. The screening and development of targeted drugs for MIRI mainly involve using methods such as HTS, proteomics, and gene knockout to identify key molecular targets related to MIRI, including enzymes associated with oxidative stress, inflammatory mediators, and proteins related to apoptosis. Subsequently, their functions are verified through cell and animal models.^[13,14]^ Based on these targets, high-throughput screening technologies, such as compound library screening and virtual screening, are applied, and cell/tissue culture models are used to screen compounds with potential therapeutic effects. Currently, certain progress has been made in the development of targeted drugs for MIRI. Some drugs mainly target oxidative stress injury, such as melatonin and N-acetylcysteine, which have entered the clinical trial stage. In addition, nonsteroidal anti drugs like aspirin, ibuprofen, and slow-release diclofenac for the treatment of inflammatory responses have also been used in clinical trials.^[15]^ Paclitaxel, trimetazidine, etc, have antiapoptotic effects during the process of apoptosis, but they are still in the stage of clinical research. Drugs that restore vascular endothelial dysfunction, such as prostaglandin E1 and prostacyclin, have vascular protective effects, and their effectiveness needs further investigation. However, in the process of screening and developing targeted drugs for MIRI, the issue of single-target specificity still exists. Many current targeted drugs only target a single-target, making it difficult to comprehensively cover the complex pathological mechanisms of MIRI. Moreover, some drugs show selectivity and toxicity towards MIRI and require further optimization. Additionally, different patients may respond differently to drugs, so individualized treatment is necessary. The design and evaluation methods of clinical trials also need further improvement to enhance the reliability and effectiveness of research results.

This study combined HTS datasets with bioinformatics methods to explore the core disease targets of MIRI and its potential targeted drugs, and verified the pharmacological effects of the targeted drugs through biological experiments. First, the SVA and limma software packages were used to merge the GSE108940 and GSE160516 datasets and remove batch effects. Subsequently, DEGs between the sham-operated group and the MIRI group were screened in the merged dataset, and enrichment analysis of the DEGs was performed. Then, LASSO and random forest were employed to screen the differential genes, and finally, 3 target genes, *FAS*, *PPARA*, and *FGF9*, were identified. Among them, the GO analysis of the DEGs was mainly enriched in “response to interleukin-1,” “MHC protein complex,” and “chemokine activity.” In addition, the *KEGG* enrichment analysis showed that the DEGs were associated with “viral protein interaction with cytokine and cytokine receptor,” “rheumatoid arthritis,” and “Influenza A.” According to the enrichment results, in MIRI, IL-1 stimulates inflammatory signaling pathways such as NF-κB, promotes the infiltration of inflammatory cells and the release of cytokines, thereby exacerbating myocardial damage. The MHC protein complex is involved in the immune response. The MHC protein complex may participate in the regulation of immune cells in MIRI and interact with cardiomyocytes, affecting the degree of myocardial damage. Chemokines are chemical signaling molecules that can attract immune cells to the site of injury. In MIRI, the increased activity of chemokines such as C5a and IL-8 can induce the aggregation of inflammatory and immune cells, exacerbating myocardial damage.^[16–18]^ The *KEGG* enrichment results suggest that the pathway of interaction between viral proteins and cytokines and their receptors indicates that MIRI may be related to viral infection or the result of the host immune response after viral infection. The rheumatoid arthritis pathway indicates that MIRI may have the characteristics of an autoimmune response. The influenza A virus pathway suggests that viral infection can cause MIRI, especially viral myocarditis.^[19]^ These relationships suggest that MIRI may involve extensive inflammatory and immune responses and intersect with viral infections and other autoimmune diseases. The study of these pathways can help us better understand the pathophysiological mechanisms of MIRI and may provide clues for the development of new treatment strategies.

In this study, LASSO and random forest were used to screen the DEGs, and finally, 3 target genes, *FAS*, *PPARA*, and *FGF9*, were determined. The *FAS* gene is an important gene in the apoptosis signaling pathway. It encodes the *FAS* protein, which is a cell-surface death receptor. The binding of the *FAS* protein to its ligand *FASL* is a crucial step in the transmission of apoptosis signals, especially playing an important role in immune cell apoptosis and organ protection. During MIRI, apoptosis of cardiomyocytes is 1 of the important causes of myocardial damage and functional decline.^[20,21]^ Programmed cell death of cardiomyocytes is related to the *FAS*/*FASL* signaling pathway. In MIRI, the expression of the *FAS* gene may increase. The increased expression of the *FAS* protein on the surface of cardiomyocytes makes them more sensitive to *FASL*, thus easily initiating the apoptosis program when stimulated by *FASL*. Inflammatory cytokines may regulate the expression of *FASL*, but overexpression of *FASL* may exacerbate the inflammatory response and aggravate myocardial damage. Studying the role of the *FAS* gene in MIRI may provide a basis for the development of new treatment strategies. It has been found that blocking the *FASL*-*FAS* interaction may reduce cardiomyocyte apoptosis and thus alleviate myocardial damage. Therefore, the expression and function of the *FAS* gene are important molecular targets for the treatment of MIRI. How to regulate the *FAS* pathway to achieve the best myocardial protection effect may be the focus of future research. The *PPARA* gene encodes the peroxisome proliferator-activated receptor α, which is a nuclear receptor protein of the PPAR family. PPARα is a transcription factor that regulates lipid metabolism and cell signaling pathways and is the core regulator of fatty acid metabolism. After MIRI, the heart needs to reestablish energy metabolic balance, and PPARα adapts to this change in energy demand by regulating fatty acid β-oxidation. Studies have shown that PPARα activators can reduce MIRI. Activation of PPARα can inhibit the production of inflammatory mediators such as TNF-α and IL-1β, thereby reducing myocardial inflammation. It can promote fatty acid oxidation, provide the energy required by the myocardium, and reduce energy metabolic disorders. PPARα can promote the survival of cardiomyocytes and the repair of the heart, increase the expression of antioxidant enzymes such as superoxide dismutase (SOD) and glutathione peroxidase (GPx), and reduce the damage of oxidative stress to cardiomyocytes. At the same time, PPARα can inhibit cardiomyocyte apoptosis by suppressing the expression of apoptosis-related genes. PPARα improves myocardial function by regulating downstream signaling pathways such as AMP-activated protein kinase (AMPK) and PI3K/AKT. Clinically, some PPARα agonists, such as gemfibrozil, have been found to have a protective effect on patients with cardiovascular diseases. However, the efficacy and safety of PPARα agonists in the treatment of MIRI need further study.^[22,23]^ *FGF9* is a member of the FGF family, belonging to the FGF-BP subfamily. *FGF9* is involved in various physiological and pathological processes such as cell proliferation, differentiation, and migration. In cardiomyocytes, *FGF9* can promote cell proliferation and myocardial repair. After MIRI, *FGF9* may activate cardiomyocyte survival and regeneration through its receptor FGFR. *FGF9* plays a role in angiogenesis, which helps to restore blood supply to the ischemic myocardial area. After MIRI, *FGF9* may promote the formation of new blood vessels, improve local blood circulation, and reduce myocardial damage. *FGF9* can affect myocardial damage by regulating the inflammatory response. It may reduce the inflammatory response after MIRI by inhibiting the production of inflammatory mediators or regulating the recruitment of inflammatory cells. In addition, *FGF9* may protect cardiomyocytes by reducing oxidative stress and calcium overload. It may also activate downstream signaling pathways such as PI3K/AKT and mitogen-activated protein kinase, thereby reducing cardiomyocyte damage. In clinical studies, the higher the expression of *FGF9*, the higher the degree of myocardial damage after myocardial infarction. Some studies have shown that an elevated *FGF9* level may be associated with a better prognosis of MIRI.^[24,25]^

Furthermore, this study utilized multiple databases, such as TCD and TCMSP, to screen compounds targeting the disease-characteristic genes *FAS*, *PPARA*, and *FGF9* of MIRI. Finally, it was found that resveratrol and GW4064 were the compounds that commonly targeted *FAS*, *PPARA*, and *FGF9*. resveratrol is a natural polyphenolic compound that has received extensive attention in the field of cardiovascular disease research in recent years. Studies have shown that resveratrol is closely related to MIRI. It is a compound with multiple biological properties, such as antioxidant, anti-inflammatory, and antiproliferative activities, and has been proven to have a cardioprotective effect during MIRI. resveratrol can scavenge free radicals, inhibit lipid peroxidation, and reduce the damage of oxidative stress to cardiomyocytes. It can suppress the production of tumor necrosis factor−α (TNF-α), IL-1β, and IL-6, thereby alleviating the inflammatory response after MIRI.^[26,27]^ resveratrol can inhibit cell proliferation, reduce cardiomyocyte apoptosis, and maintain the quantity and function of cardiomyocytes. It can promote the expression of vascular endothelial growth factor, facilitate the formation of new blood vessels, and improve myocardial blood supply. resveratrol can regulate multiple signaling pathways such as mitogen-activated protein kinase, protein kinase B (Akt), and nuclear factor E2-related factor 2 (Nrf2) to exert its cardioprotective effect. The results of many animal experiments and clinical studies have demonstrated that resveratrol has a good cardioprotective effect in MIRI. GW4064, a selective inhibitor of vascular endothelial growth factor receptor 2 (VEGFR-2), shows a protective effect against MIRI. GW4064 is a potent inhibitor of VEGFR-2, which can specifically block the activity of VEGFR-2, thereby affecting its related signaling pathways and biological effects. VEGFR-2 plays an important role in regulating vascular permeability. By inhibiting VEGFR-2, GW4064 reduces the expression of vascular permeability factors by vascular endothelial cells and decreases vascular leakage and edema after MIRI. Studies have found that the over–activation of VEGFR-2 is associated with an increase in cardiomyocyte apoptosis. The inhibitory effect of GW4064 may reduce the apoptosis rate of cardiomyocytes after reperfusion injury by suppressing the activation of proapoptotic signaling pathways. Inhibition of VEGFR–2 contributes to the recovery of myocardial contractile function. GW4064 regulates the energy metabolism and cytoskeletal stability of cardiomyocytes, which may help improve the contractile ability after myocardial reperfusion injury. VEGFR-2 also plays an important role in the inflammatory response.^[28,29]^ GW4064 may reduce the inflammatory response after MIRI by inhibiting the production of VEGFR-2-dependent inflammatory mediators and the recruitment of inflammatory cells. Multiple animal experiments and preliminary preclinical studies support the potential application of GW4064 in the treatment of MIRI. However, the optimal treatment window, dose optimization, long-term efficacy, and safety of GW4064 still need to be verified by clinical studies. In addition, the complexity and diversity of the role of the VEGFR-2 signaling pathway in MIRI also require in-depth research to fully understand the mechanism of action and clinical application prospects of GW4064.^[30]^ GW4064, a selective inhibitor of VEGFR-2, and resveratrol have potential beneficial effects when used in combination for the prevention and treatment of cardiovascular diseases. The combination of GW4064 and resveratrol has a synergistic effect on the treatment of MIRI. resveratrol is a natural and powerful antioxidant that can scavenge free radicals and inhibit lipid peroxidation. When used in combination with GW4064, resveratrol may enhance the antioxidant effect of GW4064, thereby reducing the damage of oxidative stress to cardiomyocytes. GW4064 inhibits the activity of VEGFR-2, reducing vascular leakage and the inflammatory response. Resveratrol can upregulate the expression or increase the activity of VEGFR-2, playing a synergistic regulatory role in the VEGFR-2 signaling pathway. Resveratrol can promote the survival of cardiomyocytes and the repair of the heart, and GW4064 may promote the survival and functional recovery of cardiomyocytes by reducing cell apoptosis and improving myocardial energy metabolism. Resveratrol promotes the formation of new blood vessels by promoting the expression of vascular endothelial growth factor. The inhibitory effect of GW4064 may help maintain vascular stability and, together with resveratrol, promote angiogenesis and improve myocardial blood supply. Resveratrol can inhibit the production of inflammatory mediators and the recruitment of inflammatory cells. When used in combination with GW4064, they may synergistically inhibit the inflammatory response through different mechanisms and reduce myocardial damage. The combination of resveratrol and GW4064 may reduce the risk of cardiovascular complications through multiple mechanisms such as improving myocardial function, reducing cardiomyocyte damage, and subsiding the inflammatory response.

The results of this study indicate that both resveratrol and GW4064 can enhance the viability of cells in the MIRI cell model, and there is a synergistic effect when they are used in combination. In the MIRI cell model, the indicators of cardiomyocyte injury, such as cTnI, CK, and LDH, are significantly elevated. After the administration of resveratrol and GW4064, the contents of cTnI, CK, and LDH decrease, and the combined use of GW4064 and resveratrol shows a more pronounced effect. The significant cardioprotective effects of resveratrol and GW4064 suggest that they can serve as references for rational clinical drug use. Relevant studies have shown that resveratrol indirectly affects the expression of the *FAS* gene through the PPARγ signaling pathway. Resveratrol can act as an agonist of PPARα and directly bind to and activate the *PPARA* gene.^[31]^ Additionally, by inhibiting the activity of VEGFR-2, GW4064 may indirectly influence the expression of the *FAS* and *FGF9* genes.^[32,33]^ In cell experiments, resveratrol and GW4064 reduce the expression of the *FAS* gene and increase the protein expression of *PPARA* and *FGF9* in the MIRI cell model.

In summary, this study employed GEO datasets and machine learning algorithms to screen 3 genes, *FAS*, *PPARA*, and *FGF9*, which have potential targeted therapeutic effects on MIRI. Multiple databases were used to screen GW4064 and Resveratrol, which have targeting effects on *FAS*, *PPARA*, and *FGF9*. In both the dataset analysis and cell experiments, it was found that both GW4064 and resveratrol can downregulate the expression of *FAS* and upregulate the expression of *PPARA* and *FGF9*. Notably, the combined use of GW4064 and resveratrol shows a more pronounced effect. This provides references for the treatment and mechanism exploration in clinical settings and MIRI-related research, and promotes the research and development process of new drugs for MIRI.

## Acknowledgments

Thanks to all the authors who contributed to the study.

## Author contributions

**Conceptualization:** Qi Liu.

**Data curation:** Qi Liu.

**Resources:** Qi Liu.

**Writing – original draft:** Qi Liu.

**Methodology:** Zhonggen Tian.

**Project administration:** Zhonggen Tian.

**Supervision:** Bo Hang.

**Writing – review & editing:** Bo Hang.

**Software:** Shouhu Mi.

**Validation:** Shouhu Mi.

**Visualization:** Zhen Liang.

## References

[1] Frampton J Ortengren AR Zeitler EP. Arrhythmias after acute myocardial infarction. Yale J Biol Med. 2023;96:83–94. 37009192 10.59249/LSWK8578 PMC10052595

[2] White HD Chew DP. Acute myocardial infarction. Lancet. 2008;372:570–84. 18707987 10.1016/S0140-6736(08)61237-4

[3] Kapur NK Thayer KL Zweck E. Cardiogenic shock in the setting of acute myocardial infarction. Methodist Debakey Cardiovasc J. 2020;16:16–21. 32280413 10.14797/mdcj-16-1-16 PMC7137623

[4] Zeymer U. Diagnosis and initial management of acute myocardial infarction. MMW Fortschr Med. 2019;161:34–6. 10.1007/s15006-019-0223-3 30830611

[5] Jacoby RM Nesto RW. Acute myocardial infarction in the diabetic patient: pathophysiology, clinical course and prognosis. J Am Coll Cardiol. 1992;20:736–44. 1512357 10.1016/0735-1097(92)90033-j

[6] Algoet M Janssens S Himmelreich U. Myocardial ischemia-reperfusion injury and the influence of inflammation. Trends Cardiovasc Med. 2023;33:357–66. 35181472 10.1016/j.tcm.2022.02.005

[7] Wang K Li Y Qiang T Chen J Wang X. Role of epigenetic regulation in myocardial ischemia/reperfusion injury. Pharmacol Res. 2021;170:105743. 34182132 10.1016/j.phrs.2021.105743

[8] Tian H Zhao X Zhang Y Xia Z. Abnormalities of glucose and lipid metabolism in myocardial ischemia-reperfusion injury. Biomed Pharmacother. 2023;163:114827. 37141734 10.1016/j.biopha.2023.114827

[9] Zhou Y Liang Q Wu X. siRNA delivery against myocardial ischemia reperfusion injury mediated by reversibly camouflaged biomimetic nanocomplexes. Adv Mater. 2023;35:e2210691. 36913720 10.1002/adma.202210691

[10] Yao L He F Zhao Q. Spatial multiplexed protein profiling of cardiac ischemia-reperfusion injury. Circ Res. 2023;133:86–103. 37249015 10.1161/CIRCRESAHA.123.322620

[11] Quijada NM Hernández M Rodríguez-Lázaro D. High-throughput sequencing and food microbiology. Adv Food Nutr Res. 2020;91:275–300. 32035598 10.1016/bs.afnr.2019.10.003

[12] Dillies MA Rau A Aubert J; French StatOmique Consortium. A comprehensive evaluation of normalization methods for Illumina high-throughput RNA sequencing data analysis. Brief Bioinform. 2013;14:671–83. 22988256 10.1093/bib/bbs046

[13] Yin L Li L Gao M Qi Y Xu L Peng J. circMIRIAF aggravates myocardial ischemia-reperfusion injury via targeting miR-544/WDR12 axis. Redox Biol. 2024;73:103175. 38795544 10.1016/j.redox.2024.103175 PMC11140810

[14] Li CJ Ma XP Nie H. Mechanism of Shenxiong Glucose Injection against myocardial ischemia-reperfusion injury based on network pharmacology and molecular docking. Zhongguo Zhong Yao Za Zhi. 2022;47:2759–66. 35718496 10.19540/j.cnki.cjcmm.20211108.401

[15] Yang L Jian Y Zhang ZY. Network-pharmacology-based research on protective effects and underlying mechanism of Shuxin decoction against myocardial ischemia/reperfusion injury with diabetes. World J Diabetes. 2023;14:1057–76. 37547579 10.4239/wjd.v14.i7.1057 PMC10401449

[16] Weber A Wasiliew P Kracht M. Interleukin-1 (IL-1) pathway. Sci Signal. 2010;3:cm1. 20086235 10.1126/scisignal.3105cm1

[17] Baeuerle PA Wesche H. T-cell-engaging antibodies for the treatment of solid tumors: challenges and opportunities. Curr Opin Oncol. 2022;34:552–8. 35880455 10.1097/CCO.0000000000000869 PMC9415207

[18] Colombo M Moita C van Niel G. Analysis of ESCRT functions in exosome biogenesis, composition and secretion highlights the heterogeneity of extracellular vesicles. J Cell Sci. 2013;126(Pt 24):5553–65. 24105262 10.1242/jcs.128868

[19] Liberale L Bonaventura A Montecucco F. T-cells in myocardial infarction: culprit instigators or mere effectors? World J Cardiol. 2018;10:123–6. 30386489 10.4330/wjc.v10.i10.123 PMC6205846

[20] Moulian N Berrih-Aknin S. Fas/APO-1/CD95 in health and autoimmune disease: thymic and peripheral aspects. Semin Immunol. 1998;10:449–56. 9826578 10.1006/smim.1998.0155

[21] Kiba T Saito S Numata K Sekihara H. Fas (APO-1/CD95) mRNA is down-regulated in liver regeneration after hepatectomy in rats. J Gastroenterol. 2000;35:34–8. 10632538 10.1007/pl00009973

[22] Luo R Su L-Y Li G.. Activation of PPARA-mediated autophagy reduces Alzheimer disease-like pathology and cognitive decline in a murine model. Autophagy. 10.1080/15548627.2019.1596488 PMC6984507 30898012

[23] Lee YH Jang HJ Kim S. Hepatic MIR20B promotes nonalcoholic fatty liver disease by suppressing PPARA. Elife. 2021;10:e70472. 34964438 10.7554/eLife.70472 PMC8758141

[24] Zhang L Zhang Q Teng D. FGF9 recruits β-catenin to increase hepatic ECM synthesis and promote NASH-Driven HCC. Adv Sci (Weinh). 2023;10:e2301166. 37566761 10.1002/advs.202301166 PMC10558677

[25] Sun Y Ying X Li R. FGF9 promotes expression of HAS2 in palatal elevation via the Wnt/β-Catenin/TCF7L2 pathway. Biomolecules. 2022;12:1639. 36358989 10.3390/biom12111639 PMC9687196

[26] Breuss JM Atanasov AG Uhrin P. Resveratrol and its effects on the vascular system. Int J Mol Sci . 2019;20:1523. 30934670 10.3390/ijms20071523 PMC6479680

[27] Zhou DD Luo M Huang SY. Effects and mechanisms of resveratrol on aging and age-related diseases. Oxid Med Cell Longev. 2021;2021:9932218. 34336123 10.1155/2021/9932218 PMC8289612

[28] Liu J Liu C Gao Z. GW4064 alters gut microbiota composition and counteracts autism-associated behaviors in BTBR T+tf/J mice. Front Cell Infect Microbiol. 2022;12:911259. 35811667 10.3389/fcimb.2022.911259 PMC9257030

[29] Guo J Zheng J Mu M. GW4064 enhances the chemosensitivity of colorectal cancer to oxaliplatin by inducing pyroptosis. Biochem Biophys Res Commun. 2021;548:60–6. 33631675 10.1016/j.bbrc.2021.02.043

[30] Xia Y Zhang F Zhao S. Adiponectin determines farnesoid X receptor agonism-mediated cardioprotection against post-infarction remodelling and dysfunction. Cardiovasc Res. 2018;114:1335–49. 29668847 10.1093/cvr/cvy093

[31] Okudaira N Ishizaka Y Tamamori-Adachi M. Resveratrol blocks retrotransposition of LINE-1 through PPAR α and sirtuin-6. Sci Rep. 2022;12:7772. 35546166 10.1038/s41598-022-11761-0 PMC9095727

[32] Hao H Cao L Jiang C. Farnesoid X receptor regulation of the NLRP3 inflammasome underlies cholestasis-associated sepsis. Cell Metab. 2017;25:856–67.e5. 28380377 10.1016/j.cmet.2017.03.007 PMC6624427

[33] Lu L Jiang YX Liu XX. FXR agonist GW4064 enhances anti-PD-L1 immunotherapy in colorectal cancer. Oncoimmunology. 2023;12:2217024. 37261088 10.1080/2162402X.2023.2217024 PMC10228418

